# Current Practices and Challenges in the Management of Complex Renal Stones in Africa: A Scoping Review

**DOI:** 10.7759/cureus.61134

**Published:** 2024-05-26

**Authors:** Habeeb A Abdulrasheed, Althea O George, Petra S Ayobami-Ojo, Nwachukwu O Nwachukwu, Aisha T Ajimoti, Waleed Elsayed, Ayokunle Adenipekun, Muhammad Ali Khattak, Olanrewaju Amusat, Banan Osman

**Affiliations:** 1 Urology, University Hospitals Birmingham NHS Foundation Trust, Birmingham, GBR; 2 Surgery/Urology, The Royal London Hospital, London, GBR; 3 School of Medicine and Population Health, University of Sheffield, Sheffield, GBR; 4 Internal Medicine, St. Nicholas Hospital, Lagos, NGA; 5 Nursing, Walsall Healthcare NHS Trust, Walsall, GBR; 6 Urology, Luton and Dunstable University Hospital, Luton, GBR

**Keywords:** renal unit, percutaneous nephrolithotomy (pcnl), africa, kidney, complex renal stones, staghorn calculi

## Abstract

This study reviews the challenges and management strategies for complex renal stones in Africa. Historically viewed as infection or struvite stones, recent studies highlight diverse compositions of staghorn stones. These complex stones pose significant risks, including recurrent urinary tract infections and renal impairment. In the past, conservative management of staghorn stones was associated with high morbidity; thus, surgical intervention was necessary for complete eradication. While percutaneous nephrolithotomy (PCNL) remains the standard, it carries notable risks, leading to a shift towards minimally invasive techniques. This study reviews challenges and management practices for complex renal stones and staghorn calculi in African countries, evaluating stone-free rates and associated complications.

A scoping review of the literature, following the Preferred Reporting Items for Systematic Reviews and Meta-analysis guidelines, was performed. A systematic search was conducted in PubMed, African Journal Online (AJOL) and Google Scholar, yielding 1,101 articles, but only 11 articles satisfied the inclusion criteria.

The study included 1,513 patients with 1,582 renal units, predominantly male (67.2%) with an average age of 40.7 years. Percutaneous nephrolithotomy (PCNL) was the primary treatment for the majority (71.3%), followed by open surgery (21.9%), laparoscopic surgery (4.1%), and retrograde intrarenal surgery (RIRS) (2.7%). The stone clearance rates for PCNL, open surgery, laparoscopic pyelolithotomy, and RIRS were 82.8%, 83.7%, 100%, and 92.8%, respectively. Stone sizes ranged between 22 and 80 mm, with 66% being staghorn stones. Complication rates were highest for open surgery (30.8%) and lowest for RIRS (4.7%).

Despite PCNL being the global standard, African studies still indicate a high reliance on open surgery, likely due to healthcare infrastructure, resource availability and socioeconomic factors. Enhancing access to urological care and addressing healthcare disparities are imperative for improving staghorn stone management in Africa.

## Introduction and background

Complex renal stones refer to conditions characterised by a high renal stone burden that can lead to significant morbidity. The most common form of these complex renal stones is the staghorn calculi. Staghorn calculi are branched stones that occupy the renal pelvis and calyces. Complete staghorn stones are large stones that involve at least 80% of the entire pelvicalyceal system while partial staghorn stones fill the renal pelvis and at least two calyces. Other types of complex stones are related to abnormal anatomy, and they include multiple stones located behind an infundibular stenosis, stones in a calyceal diverticulum or stones in congenital renal abnormalities such as horseshoe kidneys [1].

Historically, staghorn calculi have been thought to be infection or struvite stones [2]. However, new studies have shown that they can consist of various compositions, including mineral stones [3]. Infection stones are formed by urease-producing bacteria in recurrent UTIs. Once urea hydrolyses to ammonia and carbon dioxide, the ammonia forms salts with cations in the resulting alkaline urine, and sediments into branched staghorn calculi. Bacteria also reduce the urine's inhibitory effect on calcium oxalate and calcium phosphate [4]. These complex stones cause obstruction and recurrent urinary tract infections that may contribute to the gradual loss of renal function in patients. The epidemiology of stone burden in Africa is limited by a lack of large-scale studies and under-reporting. In Sub-Saharan Africa, the incidence rate for urolithiasis ranges from 565 to 735 cases per 100,000 population [5].

In the past, conservative management of staghorn stones was associated with high morbidity, including rates of up to 36% for recurrent urinary tract infections, urosepsis, renal impairment, and a mortality rate of around 28%. Therefore, surgical intervention aimed at complete stone eradication is the mainstay of management [6].

Percutaneous nephrolithotomy has been recommended as the standard of care, although it is associated with complications such as sepsis and haemorrhage [7,8]. Open surgery is equally fraught with a lot of complications and is only indicated in rare conditions where other less invasive methods are impossible [9]. This has led to a shift towards minimally invasive therapies that use refined endoscopes for stone fragmentation such as retrograde intrarenal surgery (RIRS) [1].

We aim to review the management practices for complex renal stones and staghorn calculi in African countries by critically appraising the management options used, stone-free rates and complications associated with each option.

## Review

Methods

Eligibility Criteria

We included all studies that provided data on the management of staghorn calculi and other complex renal stones in Africa. Excluded from our review were review articles, meta-analyses, conference presentations, commentaries, case reports, and letters to the editors. Additionally, studies published in languages other than English or those not conducted in human populations were excluded. In cases where multiple studies involved the same patient population, we selected the most recent study.

Information Sources, Search, Selection, and Data Charting

We followed the Preferred Reporting Items for Systematic Reviews and Meta-Analyses extension for Scoping Reviews (PRISMA-ScR) checklist for our study. A comprehensive literature search was conducted using PubMed, Embase, African Journal Online (AJOL), and Google Scholar to identify relevant articles on the treatment of staghorn calculi and other complex renal stones in Africa from inception to the current date. This search was conducted on April 1, 2024. The search strategy was developed collaboratively by the authors. The search results were imported into Rayyan.ai, a systematic review software, for screening and exclusion of duplicates. Articles were selected based on their relevance to the management of complex renal stones in Africa.

Quality Appraisal of Data Sources and Result Synthesis

After the keyword search, study titles, and abstracts were screened, prioritising articles that specifically addressed staghorn calculi and/or complex renal stones in African countries. Full texts were then obtained for the remaining articles, and the inclusion and exclusion criteria were applied. Any discrepancies were discussed and resolved through mutual consensus among the authors.

Results

PRISMA Flowchart

**Figure 1:**
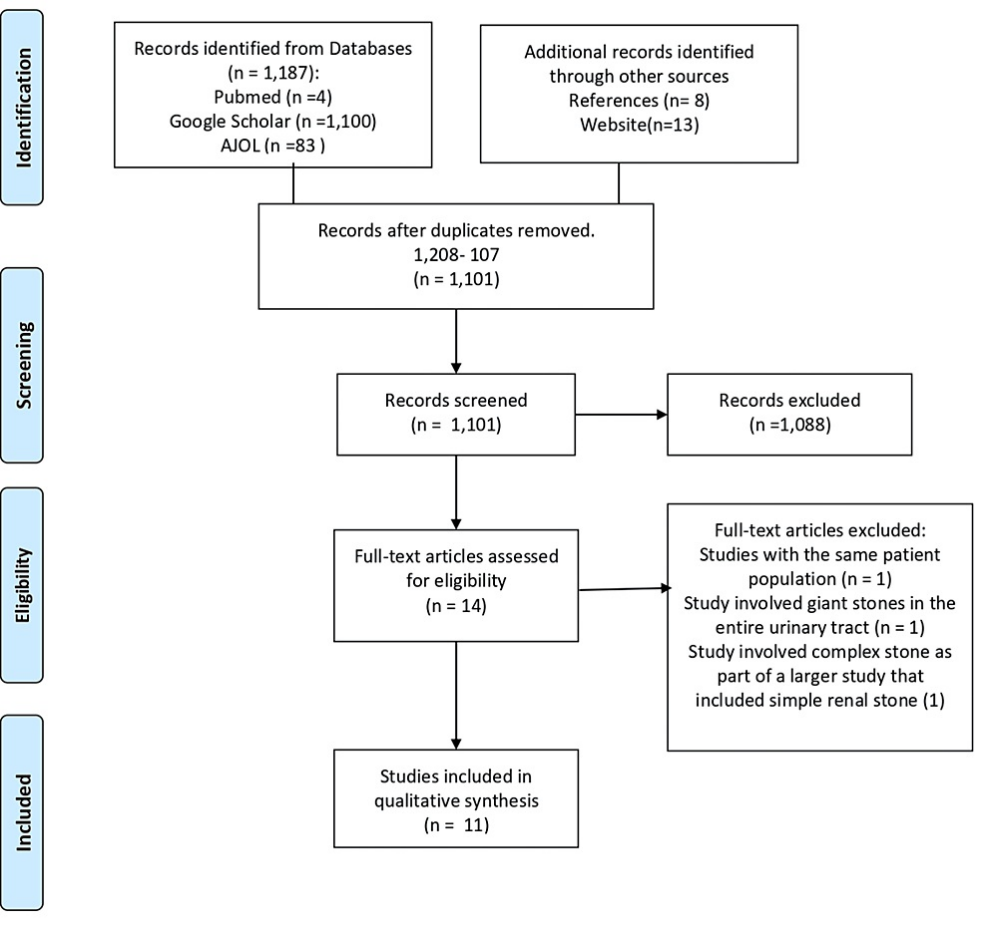
PRISMA flowchart of the study PRISMA: Preferred Reporting Items for Systematic Reviews and Meta-Analyses; AJOL: African Journal Online. n: Number of Articles

Out of 1,101 studies screened following the initial database search and deduplication, only 14 studies met the eligibility criteria. After full-text screening, one study was excluded due to overlapping patient populations with another study [10]. Another study was excluded, as it addressed complex stones throughout the entire urinary system [11]. A third article was also excluded, as it included complex stones as part of a larger study focused on simple stones [12]. Consequently, 11 studies were included in this review. The PRISMA selection process is illustrated in the accompanying Figure 1.

Study Characteristics

Eleven studies were included in the analysis, predominantly conducted in Egypt (81.8%), with the remaining studies from Morocco and Cameroon (9.1% each). The cumulative patient count across these studies was 1,513, involving 1,582 renal units. Among the participants, 67.2% were male and 32.8% were female, with an average age of 40.7 ± 12.54 years. The studies were conducted between 1999 and 2022. Most of the studies (eight) were cohort studies (72.7%) while the remaining three studies were randomized control trials (27.3%). The study characteristics are represented in Table 1.

**Table 1:** Study characteristics PCS: Prospective Cohort Study; RCT: Randomised Control Trial; RCS: Retrospective Cohort Study; SD: Standard Deviation; n: Number

Author	Year of Publication	Country of Origin	Mean Age (years)	Male (n)	Female (n)	Sample Size (n)	Study Design
Abbas [13]	2023	Egypt	49.3	340	42	382	PCS
Elawady et al [14]	2018	Egypt	39.77	189	76	265	PCS
Alazaby et al [15]	2018	Egypt	42.69	32	10	42	PCS
El-Nasr et al [16]	2020	Egypt	43.8	23	27	50	RCT
El-Nahas et al [17]	2014	Egypt	7.3	35	6	41	RCS
Shabayek et al [18]	2022	Egypt	50.51	30	13	43	RCT
Amin et al., 2022 [19]	2022	Egypt	44.8	99	107	206	RCS
Ismail et al [20]	2008	Egypt	44.5	127	81	208	RCS
Al- Kohlany et al [21]	2005	Egypt	48.6	40	48	88	RCT
Kamadjou et al [22]	2022	Cameroon	36	38	24	62	RCS
Janane et al [23]	2013	Morocco		64	62	126	RCS
TOTAL			Average Mean=40.73, SD= ±12.54	1017	496	1513	

Stone Characteristics

**Figure 2:**
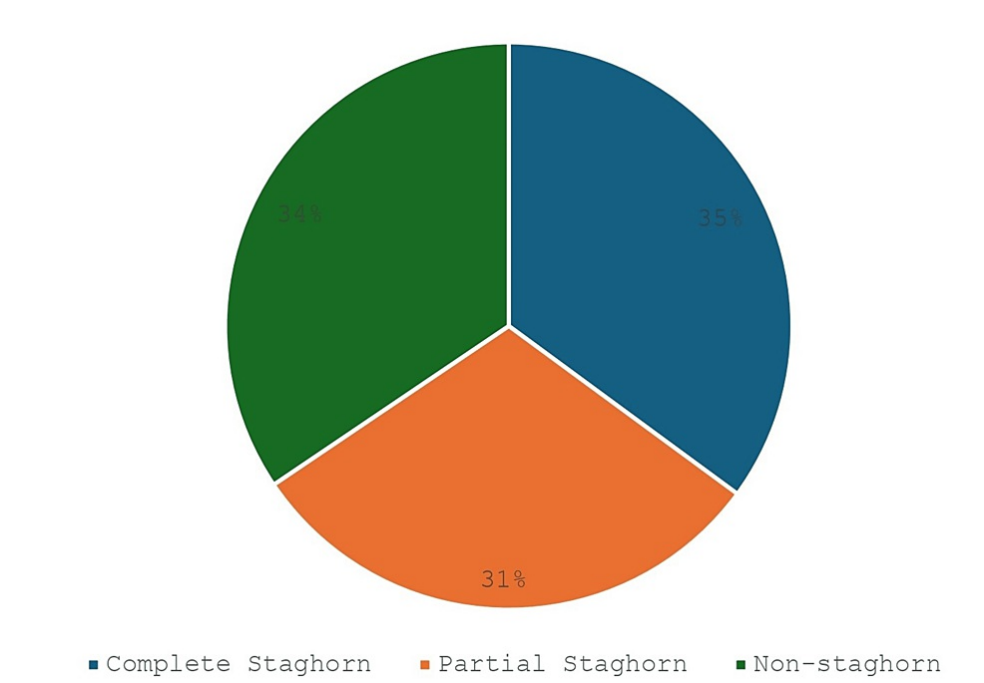
Types of renal stones and percentage representation Of all the 1582 renal units, 555 (35%) had complete staghorn stones, 482 (31%) had partial staghorn stones, and 545 (34%) had non-staghorn stones. Percentages are represented in the nearest whole number.

Our study assessed 1,582 renal systems (1442 unilateral, 71 bilateral). The stones were of various sizes, averaging 45.1 mm in diameter and ranging from 22 mm to 80 mm. Only one study described the surface area of the stone (with a mean of 872 mm^2^ ± 401.9) [13], and none of the studies discussed the volume of the stone. Complete staghorn stones were most common, identified in 555 renal units (35%). Partial staghorn stones were found in 482 units (30.5%), and multiple stones within the renal pelvis were present in 545 units (34.5%) as seen in Figure 2.

The chemical composition of the stones was detailed in a single study involving 50 cases [21]. This study revealed that uric acid was present in 14 cases (28%), calcium oxalate in 12 cases (24%), and both in six cases (12%). Additionally, struvite stones were found in 10% of the patients (five cases), cystine stones in 4% (two cases), and a combination of two or more stone types was observed in 22% of the patients (11 cases).

Clinical Presentation

Seven of the studies reviewed reported the clinical presentation of patients with staghorn stones [13,16,17,18,20,21,22]. The commonest clinical presentation seen was Urinary Tract Infection (UTI), reported in all seven studies. Other presenting symptoms were abdominal pain, urinary colic and, haematuria [22].

Preoperative Assessment

The preoperative assessment was consistent across all the articles examined, encompassing history taking, physical examination, and a range of investigations. The blood workup comprised a full blood count, urea and electrolytes, and clotting profile. Nine studies used plain CT in renal stone diagnosis in addition to plain radiograph and ultrasound KUB while the other two studies [19,23] did not specify imaging modalities. In five studies [13,16,17,20,22], contrast studies like intravenous urography and CT urography were used to assess for any urinary tract abnormalities. Urine microscopy, sensitivity, and culture were used to detect ongoing urinary tract infections, with affected participants receiving antibiotic treatment prior to further interventions.

Treatment Modalities

In our review, four treatment modalities were identified for complex renal stones, all of which were surgical interventions [13-23]. These included percutaneous nephrolithotomy (PCNL), open surgery, laparoscopic surgery, and retrograde intrarenal surgery (RIRS). Among the 1,513 patients (1582 renal units) managed for complex renal stones, PCNL and open surgery were the most frequently utilised treatment modalities, accounting for 71% and 22%, respectively. Auxiliary methods were introduced in 214 patients (14%) to enhance stone clearance rates. Shock wave lithotripsy (SWL) was the predominant auxiliary procedure, accounting for 90%, followed by flexible ureterorenoscopy (FUR) at 10%.

Nine out of the 11 reviewed articles employed PCNL as the primary treatment modality for staghorn calculi and other complex stones, representing 72% (1,089 patients, 1120 renal units) of the total patients reviewed [13,14,16-21,23]. Of these PCNL procedures, a single tract was utilised in 30.2% (329 cases) of patients, double tracts in 25.6% (279 cases), three or more tracts in 18.4% (200 cases), and the number of tracts not specified in 25.8% (281 cases). The stone-free rate following PCNL was 82.8%.

Open surgery was performed in five studies accounting for 21.1 % of patients (320 cases, 358 renal units) [15,16,19,20,22]. Various surgical techniques were applied, including open pyelotomy, nephrolithotomy, and pyelonephrolithotomy, with four patients requiring nephrectomy due to their stones. The stone-free rate for these open surgeries was 83.7%.

**Figure 3:**
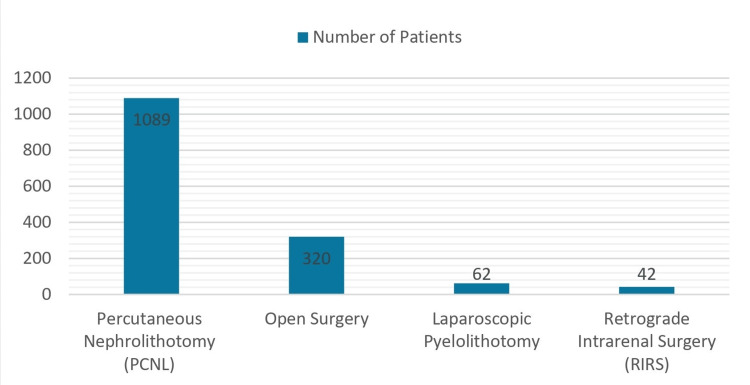
Types of surgical interventions All 1513 patients underwent surgical management for complex renal stones. Among them, 1089 (72%) underwent percutaneous nephrolithotomy, 320 (21.1%) underwent open surgery, 62 (4.1%) underwent laparoscopic pyelolithotomy, and 42 (2.8%) underwent retrograde intrarenal surgery.

Laparoscopic pyelolithotomy was employed in one study for stone treatment in 62 patients (4.1%), achieving a 100% stone-free rate [22]. Retrograde intrarenal surgery (RIRS) on the other hand was utilised in one study involving 42 patients (2.8%), demonstrating a stone-free rate of 92.8% without requiring any auxiliary procedures [15]. The summary of treatment modalities is represented in Figure 3.

Postoperative Management

Postoperatively, patients underwent routine blood tests, including haemoglobin and renal function tests. Those with suspected urinary tract infections had urine cultures performed and antibiotics given. To assess stone clearance, most studies utilized plain kidney, ureter and bladder (KUB) ultrasound between postoperative days 1 and 14 [13,14,16,20,21], with non-contrast CT requested thereafter if a radiolucent stone was suspected. However, three studies directly opted for non-contrast CT to assess stone clearance [15,17,18]. A stone larger than 4mm detected postoperatively was recorded as indicative of a failed procedure. The follow-up period varied across studies, ranging from a minimum of two months [16] to a maximum of 66 months after surgery [23].

Complications

**Figure 4:**
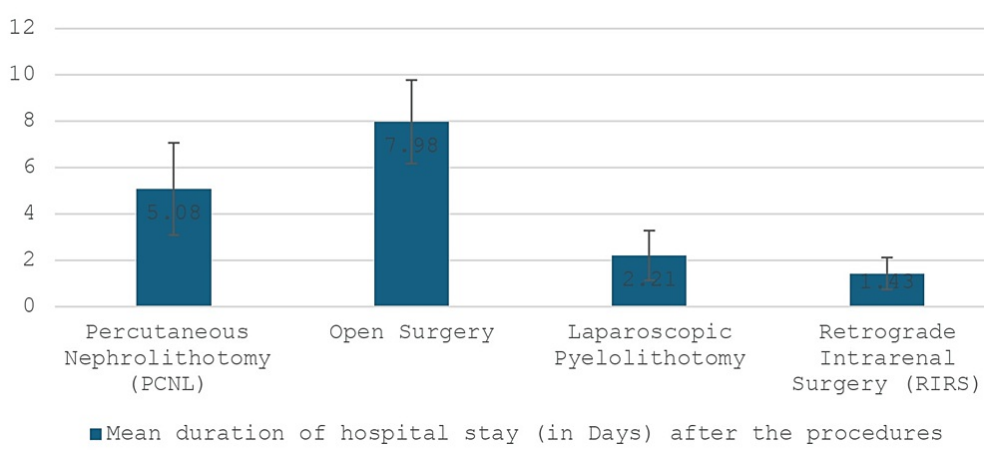
Average duration of hospital stay (in days) The average duration of each type of procedure for renal stones, along with their respective standard errors, is represented in the figure.

In this review, 22.27% (340 patients) experienced complications. The most common complications were bleeding, which manifested as haematuria observed in 8.8% (134 patients), followed by infection in 4.4% (67 patients) and urinary leakage in 2.5% (38 patients). More details are given in Table 2. The complication rates differed significantly among the surgical approaches. Open surgery had the highest rate at 30.75% (104 patients), followed by PCNL at 20.9% (227 patients), laparoscopic surgery at 16.63% (six patients), and RIRS at 4.8% (three patients). Six of the studies reported an average hospital stay of 5.08 days for patients undergoing the PCNL procedure [13,16-18,20,21] compared to 7.98 days, 2.21 days, and 1.43 days for those who had open surgery, laparoscopic pyelolithotomy, and RIRS, respectively (Figure 4). No mortality was recorded in any of the studies.

**Table 2:** Complications from all treatment modalities PCNL: Percutaneous Nephrolithotomy; RIRS: Retrograde Intrarenal Study The number of complications for each type of procedure is represented in the table, with each complication expressed as a percentage of the total number of patients with complications (340 patients).

Procedures	PCNL		Open Surgery		Laparoscopic Pyelolithotomy		RIRS		Total	
Complications	Number of Complications	Complication Rate	Number of Complications	Complication Rate	Number of Complications	Complication Rate	Number of Complications	Complication Rate	Number of Complications	Complication Rate
Bleeding	77	7.07%	55	16.42%	2	4.76%	0	0%	134	8.76%
Perforation	30	2.75%	0	0%	1	2.38%	0	0%	31	2.03%
Infection	44	4.04%	20	5.97%	3	7.14%	0	0%	67	4.38%
Leak	27	2.45%	11	3.28%	0	0%	1	1.6%	38	2.55%
Colon Injury	7	0.64%	1	0.30%	0	0%	0	0%	8	0.52%
Arteriovenous Fistula	10	0.92%	1	0.30%	0	0%	0	0%	11	0.72%
Pelvis Injury	16	1.47%	0	0%	0	0%	0	0%	16	1.05%
Pleural Injury	2	0.18%	15	4.48%	0	0%	0	0%	17	1.11%
Stent Symptoms	1	0.09%	0	0%	0	0%	2	3.2%	3	0.1%
Ileus	2	0.18%	0	0%	0	0%	0	0%	2	0.13%
Non-surgical	2	0.18%	0	0%	0	0%	0	0%	2	0.13%
Hernia	0	0%	1	0%	0	0%	0	0%	1	0.07%
Stone Recurrence	10	0.92%	0	0%	0	0%	0	0%	10	0.65%
TOTAL	227	20.89%	104	30.75%	6	16.63%	3	4.8%	340	22.27%

Discussion

Study Characteristics

We reviewed 1,513 patients over 23 years (1999-2022) from 11 studies in Africa. The ratio between females and males in the studies was 2.05:1. Urolithiasis has been reported in many studies as a male-predominant disease. However, staghorn calculi have shown a variable gender ratio [24], with females more likely to have struvite staghorn due to their predisposition to recurrent urinary tract infections [8]. The mean age of patients in this review was 40.7 years. A similar study also reported a similar middle age bracket, with patients having metabolic staghorn stones at around 55 years and those with infection stones at around 50 years [3].

Stone Characteristics

The average size of stones in our review was 45.1 mm with stone sizes ranging between 22 and 80 mm. This large size of stone informed the choice of PCNL in most of the studies, and this is the intervention of choice in stones greater than 20mm according to the European Association of Urologists recommendation on staghorn stone management [25]. Only one study analysed the chemical composition of stones with the majority being metabolic stones. This agrees with recent studies on the changing composition of staghorn calculi from infection stone to metabolic stone [3,24].

Clinical Presentation

Urinary tract infections were the most common clinical presentations seen among the eleven studies reviewed. It is not surprising as staghorn calculi were historically thought to always be caused by urinary tract infections with urease-producing bacteria [3]. This is in contrast with a study on staghorn calculi conducted in a Japanese population which found that among the 76.8% of symptomatic patients, only 14.6% exhibited features of urinary tract infection, 35% experienced vague symptoms with some pain, and 28% presented with haematuria [24]. This contrasting finding may be due to a demographic difference in the study population and a difference in health-seeking behaviour. While infection stones are still predominant in developing countries, developed countries experience more metabolic stones which may be traced to diet [8].

Preoperative Assessment

Patient preparation was similar across the studies, and also similar to that described in most studies and the guideline for managing complex renal stones. This includes initial evaluation through history taking and physical examination, laboratory investigation including biochemistry, urine culture, and infection markers [7]. The imaging modality recommended is plain CTKUB, which was done in most of the patients [9]. Patients with an ongoing urinary infection were first treated with antibiotics before any procedure, and this was the practice in all the articles reviewed.

Treatment Modalities

The primary treatment goal is to achieve complete stone removal. PCNL has been established as the gold standard for treating staghorn calculi due to its high stone-free rates and low complication rates [7,9]. Shock wave lithotripsy (SWL), ureteroscopy, or a combination of both can be employed either as primary treatment or auxiliary methods [9]. In rare instances, more invasive approaches such as open surgery or laparoscopic/robotic-assisted stone surgery may be required.

Our analysis of the reviewed articles revealed that PCNL was the most commonly used procedure for treating complex stones and staghorn calculi (72%) [13,14,16-21,23], followed by open surgery (21.1%) [16,17,20,21,23]. The significant use of open surgery may be attributed to the cost implications of less invasive procedures and the expertise of surgeons in certain African countries.

The stone clearance rates for PCNL, open surgery, laparoscopic pyelolithotomy, and RIRS were 82.8%, 83.7%, 100%, and 92.8%, respectively. However, while PCNL and open surgery were used for large stones and staghorn, the other two procedures were used in studies with relatively smaller stones. In a study that analysed the outcome of PCNL, laparoscopic, and open anatrophic nephrolithotomy for the management of large staghorn renal stone, PCNL had the lowest stone-free rate at 43.75% while laparoscopic and open surgery achieved a stone-free rate of 80% and 92.85%, respectively [26]. Although the stone-free rate of PCNL is usually lower than open in the initial postoperative period, this usually improves after auxiliary procedures like SWL and FUR [8]. A study comparing open surgery to PCNL in managing staghorn stones found that open surgery had a better initial stone-free rate but both methods achieved similar rates after additional procedures [27]. PCNL, however, offered the advantages of lower complications, shorter operative and hospitalisation times, and less blood loss than open surgery.

Postoperative Management

Routine blood tests, such as complete blood count and creatinine, are important postoperative investigations [7] and these tests were carried out in all the studies reviewed. While chest radiographs are typically performed postoperatively to rule out complications like pneumothorax or hydrothorax, this was only carried out in one study [18].

Most patients underwent follow-up imaging with KUB X-ray and ultrasound (US), and in some cases, a CT scan. The European Association of Urology (EAU) recommends KUB X-ray and/or US for stone follow-up, depending on the characteristics of the stone. A CT scan is recommended for symptomatic patients. The timing of imaging and the decision regarding the treatment of stone fragments should be based on clinician judgment in collaboration with patient preferences [25]. Early imaging within 4 weeks may lead to over-treatment.

Complications

Our review revealed that open surgery was associated with more complications (49.8% vs. 18%) compared to other modalities of treatment. The hospital stay duration was also higher in those who had open surgery (7.98 days) compared to other modalities (Figure 4). A study carried out in Taiwan, comparing PCNL to open surgery for staghorn stone management, found that there were more complications with open surgery (45.5%) when compared with PCNL (41%), however, the difference was not statistically significant [28].

Challenges of Renal Stone Management in Africa

In Africa, managing renal stones poses significant challenges due to economic constraints, limited healthcare infrastructure, and educational gaps [29]. Many cannot afford essential diagnostic tests and treatments, while underfunded hospitals, especially in rural areas, lack the necessary equipment and expertise to manage complex renal stones. Additionally, urological training programs in most regions have minimal endoscopic training capacities, prompting many professionals to seek training abroad [30]. Research on African peculiarities in the management of complex renal stones is lacking in most African countries, as evidenced by our search results, which yielded limited studies on the management of complex renal stones and staghorn calculi in Africa, with the majority of the available studies originate from North Africa.

While extensive literature reviews exist on this subject in many developed and developing countries, our search found a paucity of such research in African populations. This study is unique, as it focuses on African literature, highlighting current practices and challenges in the management of complex renal stones. The limitations of our study include the small sample size, incomplete data in some studies, potential exclusion of relevant non-English articles, and a population skewed toward Northern African countries due to study criteria. We encourage further impactful studies across other African subregions to enhance understanding and develop region-specific guidelines for managing patients with complex renal stones.

## Conclusions

This study sought to provide a comprehensive review of the management of complex renal stones and staghorn calculi in Africa, highlighting prevalent challenges and the effectiveness of various surgical interventions. It emphasizes the dominance of percutaneous nephrolithotomy (PCNL) as the primary treatment modality in Africa. To improve surgical outcomes and reduce complication rates, African clinicians need to adopt more minimally invasive techniques such as laparoscopic and laser surgeries where possible.

Overall, while the principles of staghorn stone management remain consistent globally, their implementation in Africa may be influenced by contextual factors related to healthcare infrastructure, resource availability, financial constraints, and sociocultural norms. Efforts to improve access to urological care, strengthen healthcare systems, and address socioeconomic disparities are essential for optimizing the management of staghorn stones and improving patient outcomes in Africa.
